# Bibliometric and Visualized Analysis of 2011–2020 Publications on Physical Activity Therapy for Diabetes

**DOI:** 10.3389/fmed.2022.807411

**Published:** 2022-04-07

**Authors:** Keke Huang, Jing Zhu, Shaozhe Xu, Rong Zhu, Xi Chen

**Affiliations:** ^1^School of Sports Science, Wenzhou Medical University, Wenzhou, China; ^2^School of the First Clinical Medical Sciences, Wenzhou Medical University, Wenzhou, China

**Keywords:** physical activity, diabetes, exercise, bibliometric analysis, CiteSpace

## Abstract

**Background:**

This study was designed to investigate the global emerging trends of physical activity therapy for diabetes based on a bibliometric analysis of the publications.

**Methods:**

Publication papers from 2011 to 2020 were retrieved from the database of “Web of Science Core Collection” with the topic search. A number of papers, citations, authors, countries, institutions, and references were extracted. CiteSpace was used to analyze co-citation on authors, collaborations between countries and institutions, and detect the emerging trends of burst keywords and references.

**Results:**

A total of 2651 publications were recruited in this study and showed an upward trend of annual publications. Diabetes obesity & metabolism (journal), the United States (country), Harvard University (institution), and Kaku K (author) published the most papers in this research field. “Impaired glucose tolerance” (2011–2012) was the highest strength burst keyword, while “cardiovascular outcome” (2017–2020) was the most burst keyword in the last 5 years. Moreover, “Standards of medical care in diabetes – 2014” was the strongest burst reference.

**Conclusion:**

“Physical activity therapy for diabetes” has been accepted remarkably over the last 10 years. The keywords of “impaired glucose tolerance,” “Cardiovascular outcome,” “improves glycemic control,” “Self-management,” and exercise type including “Aerobic exercise, muscle strength” may be the latest research frontiers.

## Introduction

Diabetes, also known as diabetes mellitus (DM), is a progressive and chronic metabolic disease that threatens health all over the world (1). Total or partial loss of insulin secretion or reduced insulin activity due to dysfunction of pancreatic-islet-β cell is a critical factor in the pathology of DM. There are four classes of DM (including Type 1 diabetes mellitus, Type 2 diabetes mellitus, secondary diabetes mellitus, and gestational diabetes mellitus). Type 2 diabetes mellitus (T2DM) is the most predominant type, which accounted for more than 90% (2). Previous studies revealed that T2DM was related to cardiovascular diseases (CVD), blindness, kidney failure, periodontal disease, cancer, and even death (3, 4). In recent years, numerous studies have found that physical activities, as a relatively simple, efficient, and economical approach, can alleviate the DM syndrome to a large extent (5–7).

Bibliometric is a method that combines retrieval and statistics to quantitatively analyze the changeable information and indicators in the specific domain of literature (8). The results can help researchers to better understand the hot topics and potential future research directions in a specific field, which in turn can help researchers develop new topics (9). Also, bibliometric analysis has been applied to the research fields of artificial intelligence (AI), industry, medicine, etc. (10–12). Previous studies demonstrated the bibliometric analysis of Tai Chi for health and well-being (13, 14), and others also studied bibliometric analysis of Qigong, including for diabetes (15). However, there was no study focused on the topic of bibliometric analysis of the trends of physical activity therapy or exercise (including all the exercise types) for diabetes.

Therefore, the present study aims to conduct a bibliometric analysis of physical activity therapy for diabetes which was published from 2011 to 2020 to gain new insights to guide future research and applications for diabetes prevention and treatment.

## Materials and Methods

### Source of Data and Search Strategy

A total of 4,043 published papers were collected from the Web of Science Core Collection (WoSCC) in April 2021 through the topics of “(diabetes) and (physical activity therapy),” “(diabetes) and (exercise therapy),” or “(diabetes) and (sports therapy)” under the category of the Science Citation Index Expanded (SCI-EXPANDED), Social Sciences Citation Index (SSCI), Arts & Humanities Citation Index (A&SCI), Conference Proceedings Citation Index Science (CPCI-S), Conference Proceedings Citation Index-Social Science & Humanities (CPCI-SSH), Book Citation Index–Science (BKCI-S), Book Citation Index-Social Sciences & Humanities (BKCI-SSH), and Emerging Sources Citation Index (ESCI). The retrieval strategy term was as follows: TS = [(diabetes) AND (physical activity therapy)] OR TS = [(diabetes) and (exercise therapy)] OR TS = [(diabetes) and (sports therapy)]. The publication date range is limited from 2011 to 2020, which represents the latest trends of this research field.

### Inclusion Criteria

The document types were limited to only articles and reviews. The complete records and references of all the recruited publications were downloaded for further analysis.

### Analytical Methods

#### Web of Science Core Collection

Web of Science Core Collection (WOSCC) on the website also provides the basic features of search results, such as the number of papers, citations, authors, countries, references, etc. Therefore, we used WOSCC for the primary analysis of the trend of publication features of physical activity therapy for diabetes.

#### CiteSpace

CiteSpace is a good option visual analytic tool for bibliometric analysis. Based on the relevant bibliographic publications acquired from the WOSCC (16, 17), it is used to analyze and visualize the network of one knowledge sector (18). It provides a stage for improving the reproducibility, efficiency, and exploitability of the bibliometric analysis, displaying the most representative published papers (19). In this study, CiteSpace V (version V5.7.R2) was used to analyze institutions, countries, keywords, and references in clusters and to make network maps, in which the size of the circle represents the contribution and the number of lines represents the extent of relationships. Additionally, it was also performed to analyze the citation burst of keywords and references, which provided an indication of emerging trends.

## Results

### Publication and Citation Count

**FIGURE 1:**
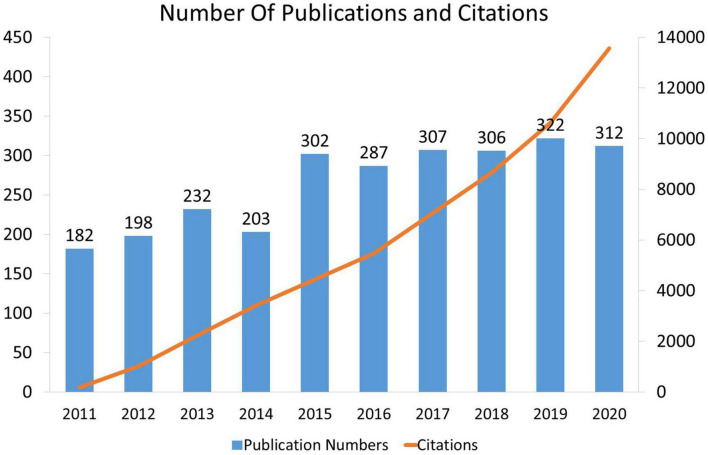
Overview research publications and citation count of physical activity therapy for diabetes from 2011 to 2020.

A total 2,651 publications were recruited in this analysis from the primary research on the WOSCC database. Among these papers, original articles accounted for 72.50% and reviews for 27.50% (Table 1). Over the past 10 years, the distribution of annual publications generally showed a steady and fluctuating upward trend. It can be roughly divided into two parts: from 2011 to 2014, the average number of publications was 203.75, while from 2015 to 2020, the trend of publication increased and the average number of publications was 303.83. The largest increase was from 2014 to 2015. Besides, the frequency of citations of these publications from 2011 to 2020 had shown a continuous upward trend in the number of folds during this period (Figure 1).

**TABLE 1:** The total publications in physical activity therapy for diabetes research.

Type	Count	Percentage (%)
Article	1,922	72.50
Review	729	27.50

### Countries and Institutes

**FIGURE 2:**
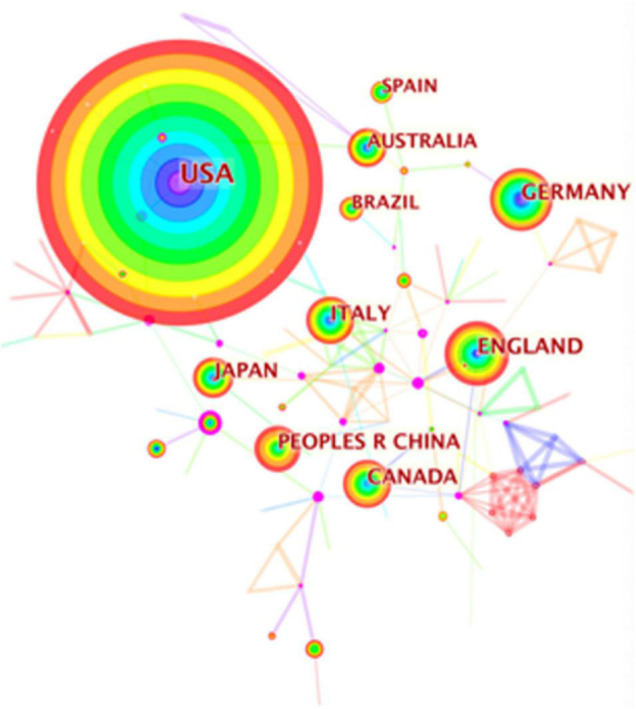
Network map of countries contributed to physical activity therapy for diabetes research. Each colored solid circle represents a country/region, and the lines between them represent collaborative relationships.

**FIGURE 3:**
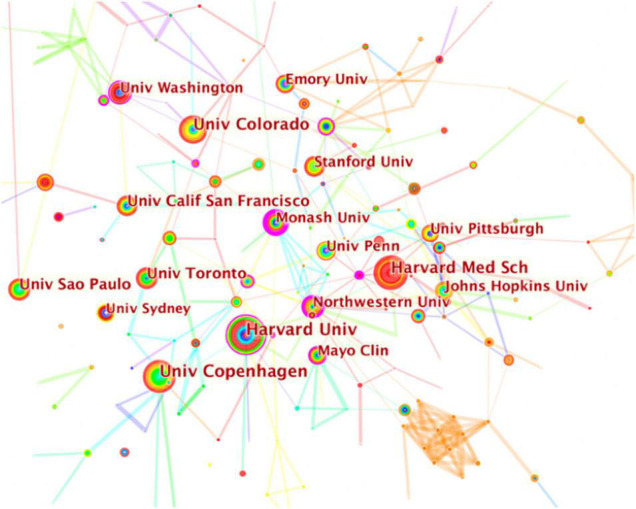
Network map of institutions contributed to physical activity therapy for diabetes research. Each colored solid circle represents a country/region, and the lines between them represent collaborative relationships.

Web of Science Core Collection provided the basic features of the recruited publications. including countries and institutes. Table 2 listed the top 10 countries that study physical activity therapy and diabetes. Americans make up the majority of the published papers (895 counts, 33.93%), the highest total citations (24,211), and H-index (75). Italy had the largest number of citations per paper (43.49), and England had the highest centrality (0.27), followed by the United States (0.22) and Italy (0.12). CiteSpace V was used to generate the network map of the countries, and the contribution is positively correlated with the size of the circle. As shown in Figure 2, the United States, Germany, and England were the top three in concentration, indicating that these three countries contribute the most. In addition, Table 3 lists the top 10 institutions that published papers related to physical activity therapy and diabetes. Harvard University (47 publications, 1.78%) is the largest contributor, followed by Copenhagen University (44 publications, 1.67%) and Harvard Medicine University (42 publications, 1.59%). As a result of the institutions’ network map, the Universities of Harvard, Copenhagen, and Harvard Medicine School are ranked in the top three in terms of concentration (Figure 3). Interestingly, the citation bursts of institutions showed that Karolinska Inst, Johns Hopkins Bloomberg Sch Publ Hlth, Vanderbilt Univ, and Univ Illinois were strongly been cited from 2017 to 2020, suggesting that these institutions are likely to contribute more to this area in the coming years.

**TABLE 2:** The top 10 prolific countries in physical activity therapy for diabetes research.

Rank	Country	Count	Percentage (%)	Centrality	H-Index	Citations per paper	Citations WoS
1	United States	895	33.93	0.22	75	27.05	24,211
2	England	229	8.68	0.27	48	33.86	7,753
3	Germany	211	8.00	0.04	38	24.34	5,135
4	Canada	177	6.71	0.04	39	33.21	5,878
5	Italy	175	6.63	0.12	35	43.49	7,611
6	China	167	6.33	0.01	21	11.68	1,950
7	Japan	157	5.95	0.03	38	32.09	4,589
8	Australia	148	5.61	0.07	36	29.24	4,327
9	Brazil	108	4.09	0.02	20	13.81	1,492
10	Spain	104	3.94	0.01	31	38.74	4,029

**TABLE 3:** The top 10 prolific institutions in physical activity therapy for diabetes research.

Rank	Institution	Count	Percentage (%)	Centrality
1	Harvard Univ	47	1.78	0.19
2	Univ Copenhagen	44	1.67	0.09
3	Harvard Med Sch	42	1.59	0.06
4	Univ Colorado	40	1.52	0.04
5	Univ Toronto	34	1.29	0.06
6	Stanford Univ	33	1.25	0.04
7	Univ Calif San Francisco	33	1.25	0.05
8	Univ Penn	32	1.21	0.04
9	Univ Washington	32	1.21	0.11
10	Univ Sao Paulo	31	1.18	0.02

### Journals

Web of Science Core Collection provided the basic features of publication journals. The top 10 journals involved in the topic of physical activity therapy and diabetes are shown in Table 4, Diabetes Obesity & Metabolism published the highest number of papers (57 counts, 2.16%), followed by Diabetes Technology & Therapeutics (35 counts, 1.33%), PLOS One(34 counts, 1.29%), and Diabetes Care(32 counts, 1.21%). While, Diabetes Care was cited 1,766 times, and was the top cited journal, followed by NEW ENGL JMED (1,420 counts), LANCET (1,257 counts), and JAMA-J AM MED ASSOC(1,210 counts). It seems that Diabetes Care is the core journal in this field (IF, 2020 = 19.11, 32 papers, 1,766 citations).

**TABLE 4:** The top 10 prolific journals in physical activity therapy for diabetes research.

Rank	Journal	Count	Percentage (%)	IF (5 years)	Cited journal	Citation count	Country
1	Diabetes Obesity & Metabolism	57	2.16	6.57	Diabetes Care	1,766	England
2	Diabetes Technology & Therapeutics	35	1.33	6.11	NEW ENGL J MED	1,420	United States
3	Plos One	34	1.29	3.24	Lancent	1,257	United States
4	Diabetes Care	32	1.21	19.11	JAMA-J AM MED ASSOC	1,210	United States
5	Current Diabetes Reports	28	1.06	4.81	Diabetologia	1,009	United States
6	Diabetologe	26	0.99	0.10	Circulation	913	Germany
7	Diabetes Research And Clinical Practice	23	0.87	5.60	Diabetes	868	Netherlands
8	Diabetic Medicine	23	0.87	4.35	J CLIN ENDOCR METAB	804	England
9	Diabetologia	22	0.83	10.12	Plos One	800	Germany
10	Current Pharmaceutical Design	21	0.80	3.11	Diabetic Med	729	United Arab Emirates

### Authors

Web of Science Core Collection provided the basic features of the authors of the publications. The top 10 authors are shown in Table 5. The greatest contributor is Kaku K(23 counts, 0.87%), followed by Riddell MC(14 counts, 0.53%), Rabasa-Lhoret R(11 counts, 0.42%), Halle M(10 counts, 0.38%), and Weitgasser R(10 counts, 0.38%). Kaku K had the highest centrality of 0.03, Riddell MC had the centrality of 0.01, and other authors’ centrality was zero, indicating that there is little connection between the authors.

**TABLE 5:** The top 10 prolific authors in physical activity therapy for diabetes research.

Rank	Author	Count	Percentage(%)	Centrality
1	KAKU K	23	0.87	0.03
2	RIDDELL MC	14	0.53	0.01
3	RABASA-LHORET R	11	0.42	0.00
4	HALLE M	10	0.38	0.00
5	WEITGASSER R	10	0.38	0.00
6	LI Y	9	0.34	0.00
7	MAAHS DM	9	0.34	0.00
8	SEINO Y	9	0.34	0.00
9	BLUMENTHAL RS	8	0.30	0.00
10	BRACKEN RM	8	0.30	0.00

### Keywords Co-occurrence and Burst Citation of Keywords

**FIGURE 4:**
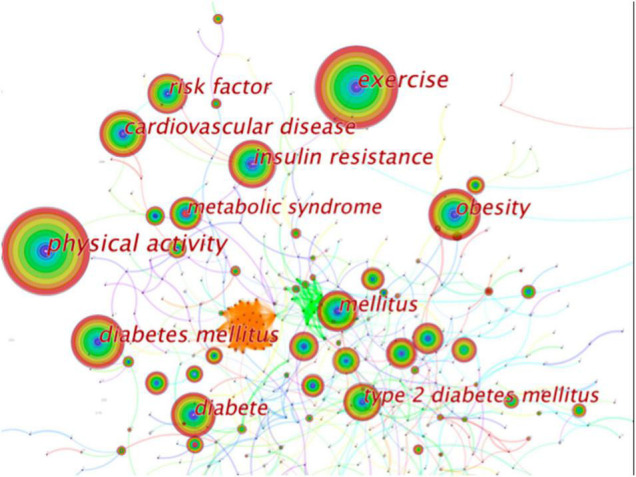
Network map of keywords contributed to physical activity therapy for diabetes research. Each colored solid circle represents a country/region, and the lines between them represent collaborative relationships.

**FIGURE 5:**
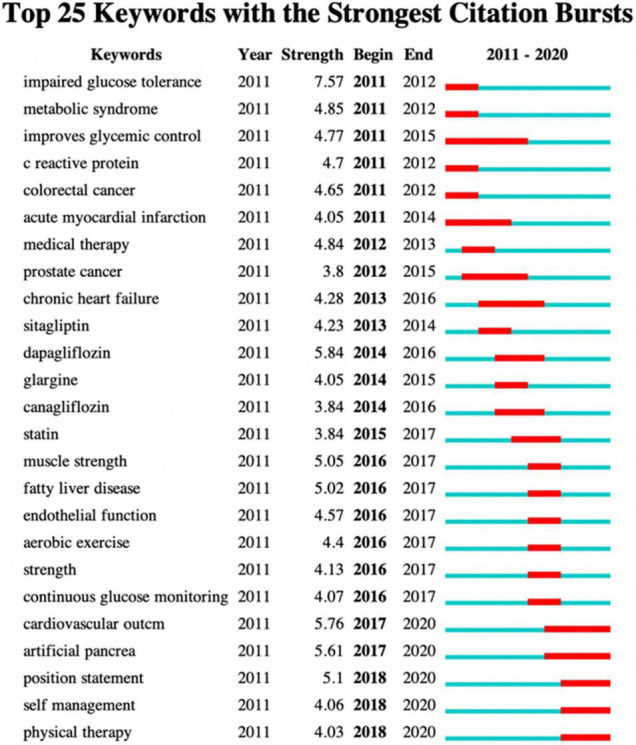
The top 25 keywords with the strongest citation bursts in physical activity therapy for diabetes research.

The network map generated by CiteSpace V showed that larger circles were “physical activity, exercise, obesity, diabetes mellitus, diabetes, insulin resistance, cardiovascular disease, metabolic syndrome” (Figure 4), suggesting that these keywords appeared more frequently and were hot keywords in the field of “physical activity therapy for diabetes.” Besides, Figure 5 gives a burst of citations of the top 25 keywords in the recent decade. The keywords of “impaired glucose tolerance, metabolic syndrome, improves glycemic control, C-reactive protein, and colorectal cancer” had been studied extensively since 2011 or earlier. However, from 2017 to 2020, some new keywords had emerged strong citations, such as “cardiovascular outcome, artificial pancrea, position statement, self management and physical therapy,” which can be extrapolated that the hot topics of these field research changed and they may also become the hot keywords in the next few years.

### Co-cited References and the Bursts Citation of the Reference

**FIGURE 6:**
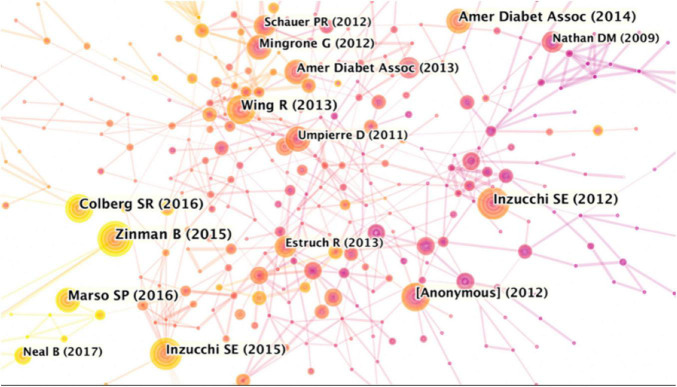
Network map of references contributed to physical activity therapy for diabetes research. Each colored solid circle represents a country/region, and the lines between them represent collaborative relationships.

As visualized in Figure 6, the network map of co-sites from CiteSpace V generated 790 nodes and 3,326 links. The top five cited papers are listed in Table 6, the most frequently cited paper was “Empagliflozin, Cardiovascular Outcomes, and Mortality in Type 2 Diabetes,” (20) which was cited 71 times, followed by “Physical Activity/Exercise and Diabetes: A Position Statement of the American Diabetes Association” (21) and “Liraglutide and Cardiovascular Outcomes in Type 2 Diabetes”(22).

**TABLE 6:** The top 5 prolific cited references in physical activity therapy for diabetes research.

Rank	Cited references	Count	Year	Centrality
1	Empagliflozin, Cardiovascular Outcomes, and Mortality in Type 2 Diabetes	71	2015	0.00
2	Physical Activity/Exercise and Diabetes: A Position Statement of the American Diabetes Association	57	2016	0.00
3	Liraglutide and Cardiovascular Outcomes in Type 2 Diabetes	55	2016	0.00
4	Management of Hyperglycemia in Type 2 Diabetes: A Patient-Centered Approach	54	2012	0.00
5	Standards of Medical Care in Diabetes 2014	53	2014	0.00

**FIGURE 7:**
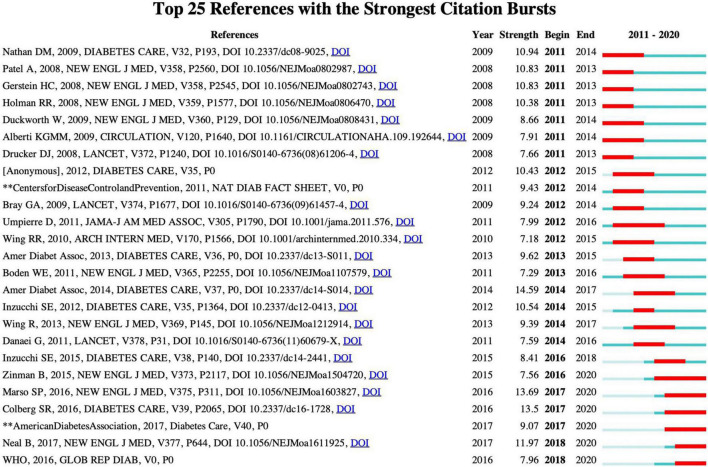
The top 25 references with the strongest citation bursts in physical activity therapy for diabetes research.

Both“Empagliflozin, Cardiovascular Outcomes, and Mortality in Type 2 Diabetes” and “Liraglutide and Cardiovascular Outcomes in Type 2 Diabetes” mainly focused on the effect of drug therapy [Empagliflozin (an inhibitor of sodium-glucose transporter 2) and Lilacropeptide (a pancreatic glucokinase 1 analog)] on cardiovascular and type 2 diabetes mellitus. While “Physical Activity/Exercise and Diabetes: A Position Statement of the American Diabetes Association” mainly focused on both aerobic and resistance training, which improved glycemic control and physical health of patients with type 2 diabetes and pre-diabetes, not only beneficial for losing weight, enhancing muscle strength, but also reducing cardiovascular hazards, increasing insulin sensitivity, and then delaying the progression of type 2 diabetes. Furthermore, Figure 7 also showed the top 25 of the burst cited literature, from which future research directions can be inferred.

## Discussion

This study collected 2,651 papers about exercise therapy (only articles and reviews) and diabetes from WOSCC publishing between 2011 and 2020, then analyzed the pieces of these literature by CiteSpace and generated the distributions of countries, institutions, journals, authors, and also analyzed the burst references and keywords, providing the researchers an overview on the research field of exercise or physical activity therapy for diabetes over the past decade and pointing out the emerging trends of these research in the future.

### The Trends of “Physical Activity Therapy for Diabetes” From 2011 to 2020

This study revealed that the number of publications slightly increased from 2011 to 2020, with most between 200 and 300 annual publications. In addition, the high publication count journals such as “Diabetes Care (IF, 2020 = 19.11), Diabetes Obesity & Metabolism (IF, 2020 = 6,57), and Diabetologia (IF, 2020 = 10.12)” had relatively high IF scores, and the top journals of medicine such as “The New England Journal of Medicine, Lancet, and JAMA-J AM MED ASSOC” also were listed as the top 5 citation counts. These results indicated that the research field of physical activity therapy for diabetes was a relatively hot topic. However, it was also challenging to publish papers in these top journals or high IF journals.

As concerned about the distribution of countries and institutions, the findings showed that the United States ranked first and far ahead in publication (895) and citation count (24,211) and H-index (75), though Italy had the most citations per paper (43.49), England had the highest Centrality (0.27). Consistent with these results, the distribution of institutions also showed that Harvard University and Harvard Medical School from the United States were listed as the top and third publication count institutions, respectively. Besides, among the top 10 publication count institutions, the United States accounts for 7 universities, suggesting that the United States had the overall influence and lead in this scientific area. However, the network map showed that these countries had little contacts or cooperation, either was the collaborations between these institutions. It seems that the lack of collaboration between different countries and institutions may contribute to the slow development of this scientific topic.

### Emerging Trends of “Physical Activity Therapy for Diabetes”

The results of the keywords network map showed that “physical activity, exercise, obesity, diabetes mellitus, diabetes, insulin resistance, cardiovascular disease, metabolic syndrome” had large circles (Figure 4), which highlighted that the research field of “physical activity therapy and diabetes” mainly focused on the effects of physical activity or exercise on improving weight management, metabolism, and reduction of insulin resistance, and then delaying the process of diabetes mellitus and reducing cardiovascular disease. In addition, Figure 5 gives a burst of citations of the top 25 keywords in the recent decade. The keywords of “impaired glucose tolerance, metabolic syndrome, improves glycemic control, C-reactive protein, colorectal cancer” had been studied extensively since 2011 or earlier. Whereas from 2017 to 2020, some new keywords had emerged as strong citations, such as “cardiovascular outcome, artificial pancrea, position statement, self management and physical therapy,” which can be extrapolated that the hot topics of these field research changed and may also become the hot keywords in the next few years.

### The Emerging Trends of Keywords and References With Citation Burst

The keywords or references with citation bursts can reflect the evolution and emerging trends of the scientific area (23, 24). Therefore, this study mainly focused on the bursts citations of keywords and references, reviewed the emerging trends of “physical activity therapy for diabetes” from 2011 to 2020 and the potential directions for future research.

### Keywords With Citation Burst

Figure 5 revealed the top 25 keywords with citation bursts. The keywords of “impaired glucose tolerance” had the highest strength (7.57). In 1997 and 2003, the diagnosis and classification of diabetes defined impaired glucose tolerance (IGT) as a condition where a person had relatively high oral glucose tolerance test (OGTT) of 140 mg/dL (7.8 mmol/L) to 199 mg/dL (11.0 mmol/L), although the glucose level had not met the criteria of diabetes (25, 26). Besides, (OGTT) of 7.8–11.0 mmol/L was also defined as prediabetes in 2020 (27). Moreover, IGT was also accepted as an essential indicator of primary prevention of type 2 diabetes mellitus (T2DM) (27). Many studies have revealed that lifestyle changes like physical activity or exercise efficacy can prevent the development of T2DM (28–30).

Secondly, the keyword of “dapagliflozin” (sodium-glucose cotransporter 2 inhibitor) had a strength of 5.84, and also other drugs like “sitagliptin” and “canagliflozin” were used to treat diabetes. Papers investigated whether the effect of exercise or physical activity combined with these drugs on the treatment of diabetes or prediabetes was better than only using these drugs (31, 32). Also, other papers compared the efficacy among these drugs or compared them with metformin for the treatment of T2DM in diabetes patients whose diabetes was inadequately controlled with diet and exercise (33–35).

“Cardiovascular outcome” also had high strength (5.76) during the last 4 years. It is well understood that diabetes is related to cardiovascular risk factors. Exercise including aerobic activity and resistance training, as well as physical activity includes all movements that increase energy expenditure and then improves glycemic control (strength 4.77), reducing cardiovascular risk factors and even the overall mortality risks in patients with diabetes (36–39). However, to avoid exercise-related cardiovascular events, ACSM recommends that normally sedentary individuals with diabetes who desire to participate in exercise should have a medical screening first (40). Whereas no evidence supports that pre-exercise medical screening for asymptomatic diabetes patients could reduce the risk of exercise-related cardiovascular events (41, 42).

Diabetes “self-management” education (DMSE) should be included for patients with diabetes to develop a plan considering the patient’s age, work or school conditions, physical activity, eating as well as emotional well-being, of which play an essential role in the improvement of health outcome with cost-saving, and should be measured and monitored as part of care (43, 44).

“Aerobic exercise” (4.4), “muscle strength” (5.05), and “strength” (4.13) had relatively high citation bursts and reflected the hot issue that “which type of exercise benefits diabetes most?” Most often, exercise is classified as aerobic or anaerobic training. Aerobic exercise training increases cardiac output, oxidative enzymes, and insulin sensitivity in patients with T2DM, e.g., regular training reduced A1C, insulin resistance, and triglycerides in patients with type 2 diabetes (45). Resistance training benefits patients with T2DM with improvements in glycemic control, insulin resistance, and body fat mass (weight management) (46). Although Church et al. demonstrated that aerobic training combined with resistance training improved HbA(1c) levels in patients with T2DM, which was better than aerobic or resistance training alone (38), it is recommended that all types of exercise including physical activity benefit patients with diabetes. Furthermore, other types of exercise such as Tai chi, yoga, and some balance training were the hot topics in the exercise or physical activity therapy for diabetes in recent years. How to provide individual-specific exercise prescriptions and achieve diabetes-related health may be recommended in future work.

### References With Citation Burst

Figure 7 demonstrates the top 25 references with the citation bursts. Among these references, we pay special attention to the highest strength reference “Standards of medical care in diabetes – 2014” (strength 14.59), which is a position statement for medical care of diabetes in 2014 (47). As concerned about the physical activity section, it recommended the adults performed moderate-intensity aerobic physical activity at least 150 min/week (50–70% maximum heart rate), with a frequency at least 3 days/week and no more than 2 consecutive days without exercise. Also, resistance training was be advised for the patients with T2DM at least twice a week. Furthermore, the reduction of calories and dietary fat intake was recommended for weight management, which contributed to reducing the risk for developing diabetes (44). Previous studies also revealed that higher intensities of exercise or adequate exercise may provide greater benefit for diabetes, and then the key issue came with how much intensity or duration was enough to benefit health? What were the results of the health response of short-time with high-intensity exercise vs. long-time with low-intensity? As high intensity or vigorous exercise had a relatively higher risk of cardiovascular events, how to balance the benefit and risk overall for health? The American Diabetes Association generally suggested that the physical activity of moderate-intensity exercise with 150 min/week and no more than 2 consecutive days without exercise for diabetes or prediabetes patients. In terms of this, exercise prescriptions should be specific for individuals (6).

The burst references in the latest years showed that Marso et al. (22), Zinman et al. (20), and Neal et al. had high citation strength. However, these papers mainly focused on the effects of drugs on diabetes.

Colberg et al. (21) published a position statement on physical activity/exercise and diabetes. This paper pointed out that maintenance of physical activity or exercise could be beneficial for the overall health, including improvement of blood glucose control, reduction of cardiovascular risk factors and body weight in patients with T2DM and prediabetes, although the key challenges related to physical activity benefits for patients with diabetes (including type I and type II) vary with exercise type, intensity, duration, frequency, and the risk of diverse events (e.g., cardiovascular events, hypoglycemia). Specific physical activity recommendations or precautions should be tailored to meet each individual’s health-related complications such as the type of diabetes, age, and activity habit. In addition, it also listed the recommendations for most situations. Furthermore, it encouraged individuals to have regular physical activity and reduce daily sedentary time, e.g., “prolonged sitting should be interrupted with bouts of light activity every 30 min.” The behavior change is considered more important in the maintenance of lifetime physical activity. Thus, “Standards of Medical Care in Diabetes – 2017” mainly refocused on life management and recommended that prolonged sedentary sitting should be interrupted with short bouts of physical activity (48).

Importantly, as “Management of Hyperglycemia in Type 2 Diabetes: A Patient-Centered Approach” said, it is crucial to combine medication, diet, exercise appropriately, but more significant is that patients should be involved in the development of treatment plans and receive exclusive treatment programs to improve the feasibility and effectiveness of disease treatment (49).

### Strength and Limitation

To the best of our knowledge, this study first reviewed the research area of physical activity or exercise therapy and diabetes from 2010 to 2020 through bibliometric analysis. The present study provides insights into the growth trends and the current key challenges which require further study of this scientific area based on the published work. Researchers can better understand the latest developments and hot topics in a particular field. Nevertheless, this study has some limitations. Firstly, the literature is searched only on the database “Web Of Science Core Collection” to ensure the quality of publications; the other related publications may be leaked, which may result in an incomplete analysis. Besides, in cluster analysis, we only showed information about the (countries, institutions, authors, and keywords) nodes, which may result in the analysis not being in place.

## Conclusion

This study provides a bibliometric analysis of “diabetes and physical therapy” from the publications over the past 10 years. Based on the quantitative analysis of this scientific area, it showed that the most influential of countries, institutes, authors, journals, respectively. The keywords of “impaired glucose tolerance,” “Cardiovascular outcome“, “improves glycemic control,” “Self-management,” exercise type including “Aerobic exercise, muscle strength” may be the latest research frontiers. Based on the citation burst, the reference “Colberg (21) may be worthy of attention.

## Author Contributions

XC supervised the whole program. KH and JZ searched the publications and performed the bibliometric analysis. SX prepared the tables and figures. KH, JZ, and XC wrote the manuscript. RZ and XC revised the manuscript. All authors approved the final manuscript.

## Conflict of Interest

The authors declare that the research was conducted in the absence of any commercial or financial relationships that could be construed as a potential conflict of interest.

## Publisher’s Note

All claims expressed in this article are solely those of the authors and do not necessarily represent those of their affiliated organizations, or those of the publisher, the editors and the reviewers. Any product that may be evaluated in this article, or claim that may be made by its manufacturer, is not guaranteed or endorsed by the publisher.
